# The Semi-columellar Strut Technique: Correction of Slight Sagittal Deviation of the Septum

**DOI:** 10.7759/cureus.108893

**Published:** 2026-05-15

**Authors:** Aleksandre Kuzanov, Ketevan Kuzanov, Ivane Kuzanov

**Affiliations:** 1 Department of Plastic and Reconstructive Surgery, Kuzanov Clinic, Tbilisi, GEO; 2 Department of Medicine, Riga Stradins University, Riga, LVA

**Keywords:** aesthetic surgery, innovations, innovations in rhinoplasty, plastic surgery, rhinoplasty, semi-columellar strut

## Abstract

Reinforcement of the nasal tip is a key step in modern rhinoplasty, typically achieved using techniques such as tongue-in-groove, septal extension grafts, or columellar struts. We present a novel method, the semi-columellar strut, designed for patients with mild sagittal septal deviation and a properly aligned caudal septum. This technique involves a partial incision of the caudal septum to increase flexibility, suturing of the alar domes, and fixation of the medial crura to the septum, followed by a tip rotation control suture securing the upper medial crura to the nasal dorsum. The semi-columellar strut provides effective tip reinforcement while maintaining independent tip projection, can be completed in 3-5 minutes, and avoids the complexity and operative time associated with traditional septal reconstruction procedures. Based on our clinical experience of 58 patients, the technique yields consistently satisfying aesthetic outcomes in appropriately selected cases. While it does not correct internal septal deviation or improve nasal airflow, it achieves reliable aesthetic outcomes in selected cases, offering a simple, efficient alternative for nasal tip stabilization in rhinoplasty.

## Introduction

Reinforcing the nasal tip is one of the most important procedures in modern rhinoplasty. The nasal tip defines facial harmony, and its long-term stability is a primary determinant of surgical success. Various methods can be used for this surgery: tongue-in-groove, septal extension graft, columellar strut and modifications of these methods [1]. Postoperative loss of nasal tip support is among the most common faults in septorhinoplasty, and controlling nasal tip rotation, broadly defined as the relationship between the base of the nose and the face in profile view, is recognised as one of the most complex aspects of the procedure [2]. Each of these methods carries distinct advantages and limitations with respect to operative complexity, operative duration, and suitability for the anatomical characteristics of individual patients.

In this article, the authors present an innovative rhinoplasty technique invented by the senior author (A.K.) - the semi-columellar strut. This method is particularly useful in patients presenting with a slight sagittal deviation of the septum, where the caudal septum is properly aligned on the anterior nasal spine.

Sagittal deviation often requires procedures such as L-strut reconstruction or extracorporeal septoplasty, both of which are technically demanding, time-consuming and surgically complex. Extracorporeal septoplasty involves subtotal resection of the septal cartilage, straightening or replacement of the L-strut, and reattachment of the septal framework at the keystone and caudal areas, and is typically reserved for cases of severe, complex caudal deformity [3]. In contrast, the semi-columellar strut offers a simpler and faster alternative, which, based on our preliminary experience, appears to achieve comparable aesthetic results with a meaningfully reduced operative burden in appropriately selected patients.

## Technical report

Required instruments

The semi-columellar strut technique requires only standard rhinoplasty instrumentation. Specifically, the procedure utilizes a scalpel, a needle holder, cartilage forceps, and 5-0 Prolene suture. No specialized grafting instruments or cartilage harvest tools are required beyond those already in use for routine rhinoplasty.

Preoperative preparation

Preoperative preparation follows the standard protocol for open structural rhinoplasty. This includes routine preoperative assessment, appropriate anaesthetic planning, and standard sterile preparation and draping. Patient selection is of particular importance: the semi-columellar strut is indicated for patients with mild sagittal septal deviation in whom the caudal septum is properly aligned on the anterior nasal spine. Patients with significant structural septal deviation - particularly those who require functional correction of the nasal airway - are not suitable candidates for this technique alone.

Surgical steps and technique

The procedure is performed as an integrated component of open rhinoplasty, following the standard transcolumellar and marginal incisions and elevation of the soft-tissue envelope. The steps of the semi-columellar strut technique are described below in sequential order.

Step 1: Partial Incision of the Caudal Septum

Using a scalpel, an incomplete incision is made in the caudal portion of the septum. Crucially, this incision does not transect the septum entirely; rather, it partially scores the cartilage, increasing its inherent flexibility without disrupting its continuity. This maneuver allows the septum to function with greater independence from the adjacent structures, providing a mobile but structurally competent midline support. By preserving septal continuity, the technique avoids the destabilizing consequences of full septal transection (Video 1).

**Video 1:** An incomplete incision in the caudal septum.

Step 2: Approximation of the Alar Domes

Following the partial septal incision, the alar domes are brought together and secured to one another using a dome-binding suture placed with the needle holder. At this stage, the septum remains structurally independent from the domes, intentionally maintained as a separate unit. This independence preserves the option for individual adjustment of tip projection in subsequent steps and prevents premature restriction of tip mobility before definitive fixation is achieved (Video 2).

**Video 2:** Suture placement bringing the alar domes together.

Step 3: Fixation of the Medial Crura to the Septum

Two sutures of 5-0 Prolene are then placed using the needle holder and cartilage forceps to secure the medial crura to the caudal septum. Careful suture placement at this stage is essential to ensure symmetrical fixation and avoid inadvertent distortion of the medial crural footplates (Video 3).

**Video 3:** Suturing of the medial crura to the septum.

Step 4: Tip Rotation Control Suture

To complete tip reinforcement and guard against postoperative tip droop, a tip rotation control suture is placed. Using the needle holder and 5-0 Prolene, the upper portion of the medial crura is secured to the nasal dorsum. This suture prevents hyper-rotation of the tip and tethers the tip complex to the stable dorsal framework. Following placement of this suture, the nasal tip is structurally reinforced and no longer susceptible to hyper-rotation; however, it remains functionally independent from the remainder of the nasal framework, allowing the surgeon to assess and adjust projection separately if required (Video 4).

**Video 4:** Tip rotation control suture.

Step 5: Final Assessment of Tip Reinforcement

Following completion of the four operative steps, the overall tip reinforcement is assessed intraoperatively. The nasal tip is evaluated for stability, projection, rotation, and symmetry. As the tip remains structurally independent from the broader nasal framework at this stage, any discrepancy in projection can be identified and addressed prior to closure (Video 5).

**Video 5:** The structurally reinforced nasal tip, which no longer hyper rotates.

Surgical Outcomes

The semi-columellar strut technique has been applied in a consecutive series of 58 patients by the senior author (A.K.). Of these, 57 patients (98.3%) reported a high degree of subjective satisfaction with their aesthetic outcome at follow-up, and objective clinical assessment confirmed concordant, satisfying nasal tip positioning, projection, and symmetry in these cases. The single adverse outcome occurred in a patient in whom the technique was applied outside its intended indication: the patient presented with significant, rather than mild, sagittal septal deviation, and the semi-columellar strut was insufficient to achieve an acceptable result. Revision surgery with extracorporeal septoplasty was subsequently required.

## Discussion

For effective nasal tip reinforcement, whether using a dorsally placed graft or a septal extension graft, it is crucial that the nasal dorsum is straight. Any deviation or curvature compromises nasal tip symmetry and the desired long-term outcome [4].

Thus, in case of a deviated nasal dorsum, septal reconstruction must be performed prior to reinforcing the nasal tip in order to straighten the septum.

The aim of correcting septal deviation may be either aesthetic (realignment of the dorsum and refinement of the tip) or functional (restoration of proper airflow through the nasal passages) [5]. It is important to emphasise that the semi-columellar strut technique does not address the internal deviation of the septum but rather camouflages it; accordingly, this procedure should be performed solely for aesthetic purposes as it does not improve nasal airflow.

The primary advantage of this technique lies in its efficiency and simplicity. In the authors' experience, the procedure can be completed in just 3 to 5 minutes, representing a meaningful reduction in operative burden relative to more extensive septal reconstruction approaches, which may require considerably longer operative time [6,7].

The authors consider this technique suitable for cases with mild sagittal deviation of the septum, particularly when the caudal part of the septum is properly positioned on the anterior nasal spine. Future prospective studies with standardised outcome measures and longer follow-up intervals would be valuable in further characterising the reproducibility and durability of this approach across centres.

## Conclusions

The semi-columellar strut is a straightforward and efficient technique for nasal tip reinforcement in patients with mild sagittal septal deviation and a well-positioned caudal septum. It provides stable tip support, preserves independent tip projection, and significantly reduces operative time compared with traditional septal reconstruction methods. In a series of 58 patients, the technique achieved objectively and subjectively satisfying outcomes in 57 cases (98.3%), with the single unsatisfactory result attributable to application outside the intended indication, highlighting the paramount importance of appropriate patient selection. This approach is particularly valuable for aesthetic rhinoplasty cases where functional correction of septal deviation is not required, offering a reliable and reproducible option for tip stabilization.
